# Acid-Base Disturbances in Patients with Asthma: A Literature Review and Comments on Their Pathophysiology

**DOI:** 10.3390/jcm8040563

**Published:** 2019-04-25

**Authors:** Ioannis Vasileiadis, Emmanouil Alevrakis, Sevasti Ampelioti, Dimitrios Vagionas, Nikoletta Rovina, Antonia Koutsoukou

**Affiliations:** 1 Intensive Care Unit, 1st Department of Respiratory Medicine, National and Kapodistrian University of Athens, Sotiria Hospital, 115 27 Athens, Greece; vagionasdimitrios@gmail.com (D.V.); nikrovina@med.uoa.gr (N.R.); koutsoukou@yahoo.gr (A.K.); 2 4th Department of Respiratory Medicine, Sotiria Hospital, 115 27 Athens, Greece; m.alevrakis@gmail.com; 3 5th Department of Respiratory Medicine, Sotiria Hospital, 115 27 Athens, Greece; sevi.ampelioti@gmail.com

**Keywords:** asthma, lactic acidosis, hyperchloremic acidosis, hypocapnia, hypercapnia

## Abstract

Asthma is a common illness throughout the world that affects the respiratory system function, i.e., a system whose operational adequacy determines the respiratory gases exchange. It is therefore expected that acute severe asthma will be associated with respiratory acid-base disorders. In addition, the resulting hypoxemia along with the circulatory compromise due to heart–lung interactions can reduce tissue oxygenation, with a particular impact on respiratory muscles that have increased energy needs due to the increased workload. Thus, anaerobic metabolism may ensue, leading to lactic acidosis. Additionally, chronic hypocapnia in asthma can cause a compensatory drop in plasma bicarbonate concentration, resulting in non-anion gap acidosis. Indeed, studies have shown that in acute severe asthma, metabolic acid-base disorders may occur, i.e., high anion gap or non-anion gap metabolic acidosis. This review briefly presents studies that have investigated acid-base disorders in asthma, with comments on their underlying pathophysiology.

## 1. Introduction

Asthma is a common yet complex airway disease, characterized mainly by chronic airway inflammation and temporal variability in symptoms and expiratory airflow limitation. A complete analysis of the pathophysiology of the disease is beyond the scope of this paper, but a brief review of the main mechanisms involved in disease exacerbations is necessary to better grasp the acid-base derangements often encountered in these patients. Although asthma is increasingly being recognized as a heterogeneous disease with many different phenotypes, the mechanics of disease exacerbation seem to be common amongst patients. In acute asthma exacerbations, exposure to a precipitating factor leads to an exaggerated inflammatory response in the airways owing to an innate airway hyperresponsiveness in these patients. The immediate result of this inflammatory response is contraction of bronchial smooth muscles, bronchial edema, and mucus hypersecretion leading to mucus plugging. The consequent narrowing of airway diameter leads to increases in airway resistance and limitation of expiratory flow, and hence to air trapping and dynamic hyperinflation. As tidal breathing then starts taking place within the flat portion of the pressure-volume curve, the elastic work of breathing is dramatically increased. Hyperinflation essentially leads to the generation of an inspiratory threshold, which is reflected by the presence of positive end-expiratory pressure (auto-PEEP). This inspiratory threshold must be overcome by the inspiratory muscles during each breath in order for inspiratory flow to begin. Another deleterious factor is the disadvantageous positioning of respiratory muscle length-tension curves in these large lung volumes, necessitating the recruitment of accessory inspiratory and expiratory muscles, further contributing to respiratory muscle fatigue [1].

The aforementioned pathophysiological mechanisms eventually lead the deteriorating patient towards ventilatory failure. Nonetheless, acute respiratory failure is a far more common event in acute asthma. Indeed, hypoxemia is widely prevalent, with PaO_2_ levels of less than 60 mmHg even in non-severe asthma [2] having being reported in several studies [3]. Even though this effect was attributed to ventilation/perfusion (V_A_/Q) inequalities owing to regional differences in airflow, it was not until the advent of the multiple inert gas elimination technique that this phenomenon was adequately demonstrated [4].

**Figure 1:**
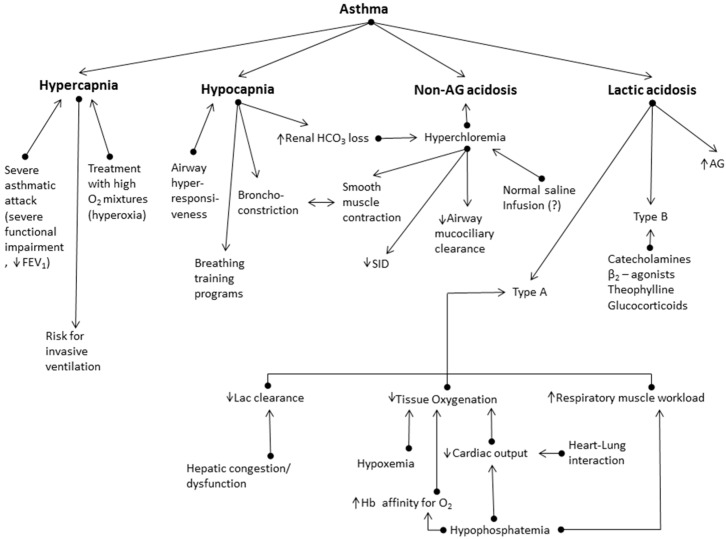
Acid-base disorders in asthma. FEV_1_ = forced expiratory volume in 1 s. AG = anion-gap.

The pathophysiological disorders in asthma result in various acid-base disturbances; these are summarized in Figure 1 and are briefly discussed below.

## 2. Respiratory Alkalosis

Acute asthmatic crisis is usually accompanied by hyperventilation and hypocapnia with respiratory alkalosis [3,5]. However, it seems that mild, asymptomatic asthma is also associated with hypocapnia. Studies that have demonstrated hypocapnia in asymptomatic asthmatics, as well as during asthmatic attacks, are presented in Table 1.

In a study, asymptomatic patients with asthma had significantly lower partial pressure of carbon dioxide (PCO_2_) in arterial blood and end-tidal PCO_2_ (P_ET_CO_2_) values compared to normal subjects, with no difference in the ventilatory pattern [6]. To note, there was no statistically significant difference in the other acid-base variables between asthma patients and healthy controls, i.e., the pH values were similar in both groups. Hypocapnia was attributed to airway hyperresponsiveness. In another study, similar results were found; normal subjects had higher P_ET_CO_2_ at rest compared to asthmatic patients. This study evaluated the effect of hypocapnia and hypercapnia in patients with asthma and in healthy subjects. It was found that while the fall of PCO_2_ increased airway resistance in asthmatic patients, it did not significantly change the respiratory resistance in normal individuals [7]. It was suggested that hypocapnia is probably associated with the airway obstruction observed in asthmatics, thus having an important role in the pathophysiology of asthma. Low PCO_2_ has been demonstrated to increase airway smooth muscle tension in animal models, as well; the proposed mechanism by which hypocapnia affects smooth muscle contraction is the alteration of calcium uptake due to an increase in intracellular pH [9,10]. Corroborating the above findings, it has further been shown that high PCO_2_ values cause bronchodilation and reduce airway resistance in both asthma patients and normal subjects [7,11]. As hypocapnia has been implicated in the pathophysiology of asthma, training techniques have been proposed to improve patients’ respiratory pattern, reduce hyperventilation, and increase PCO_2_, in an attempt to reverse the bronchoconstrictive effect of hypocapnia. Breathing retraining has been used in the management of asthma. The Buteyko breathing technique, an innovative treatment approach for asthma, named after Professor Konstantin Buteyko, has been widely applied [12]. In a recent trial, breathing training programs improved disease-related quality of life in adult asthmatic patients [13].

## 3. Respiratory Acidosis

Respiratory acidosis is a very common acid base disturbance in acute severe asthma and is widely considered to be an ominous finding. Its early recognition and treatment is important and decisive for the final outcome, as it can lead to respiratory failure and arrest if prolonged. Studies relating hypercapnia during asthmatic attacks are presented in Table 2.

In asthmatic patients, hypercapnia and respiratory acidosis occur in clinical exacerbations characterized by severe airway obstruction [14]. Simpson et al., in one of the earliest published studies, noted that hypoxemia and respiratory acidosis are common in children with acute severe asthma, as in 10 out of the 24 acute attacks that were studied CO_2_ retention was observed, while three patients developed carbon dioxide narcosis [17]. On the other hand, Weng et al. found that the degree of hypoxemia correlated with the degree of airway obstruction, but neither PaCO_2_ nor pH did. This study reported 177 events in 139 asthmatic children, both symptomatic and asymptomatic. Respiratory acidosis or mixed acidosis was present in severely dyspneic patients [18]. Lee et al. noted that PaCO_2_ was significantly higher and the arterial blood pH lower in asthmatics who died, and delays in providing mechanical ventilation led to worse outcomes [15]. Several studies have attempted to find clinical signs that could be correlated to the presence of respiratory acidosis. Cham et al. tried to find clinical predictors for acute respiratory acidosis in 141 episodes in patients with either asthma or chronic obstructive pulmonary disease (COPD). A total of 41 patients had hypercapnia (32.3%), and acidosis was present in 27 (65.9%). The frequency of acute respiratory acidosis was 0.39 in COPD and 0.10 in asthma. Drowsiness, flushing, and intercostal retractions were strong predictors for acute respiratory acidosis [16]. It has also been reported that the absence of pulsus paradoxus makes the presence of hypercapnia unlikely, although it is noted that clinical signs and symptoms during acute severe asthma often are not correlated with the severity of the functional impairment [19]. Raimondi et al. performed a case series study exploring acid-base patterns in patients admitted for acute severe asthma, in correlation with forced expiratory volume in 1 s (FEV_1_) values [8]. Airway obstruction severity did not seem to correlate significantly with PaO_2_ values, a finding also reported in several other studies in the past [18,20,21] and thought to be due to V_A_/Q mismatch not being connected to air flow rates; moreover, treatment with β-agonists may lead to further widening of V_A_/Q mismatch. A statistically significant reverse correlation was demonstrated between FEV_1_ and PaCO_2_ levels. Inability to complete spirometry was shown to be accompanied with a significantly higher frequency of respiratory acidosis, with some patients being deemed unable to undergo a spirometry maneuver by the attending physician. Less severe cases of acute asthma presented mostly with respiratory alkalosis.

Hypercapnia in asthma, in addition to the severity of the disease, is also associated with the therapeutic administration of oxygen. Thus, in patients with severe asthma exacerbation, significant increase (≥4 mmHg) in transcutaneous PCO_2_ (PtCO_2_) was observed in a higher proportion in those receiving high oxygen mixtures (>8 L/min), compared to those who received titrated oxygen (to achieve oxygen saturation of 93–95%) [22]. Hypercapnia induced by hyperoxia has been known for quite some time, and has been mainly studied in patients with COPD. For the pathophysiological interpretation of the mechanism involved, the simplistic view of Campbell (1960) had prevailed, who assumed that in chronic COPD patients with hypercapnia, respiratory control is based only on hypoxic drive as their hypercapnic respiratory drive is blunted [23]. Subsequent studies, mainly in patients with COPD, did not confirm this hypothesis. It was found that oxygen-induced hypercapnia does not indicate a deficiency/impairment of the respiratory control system for CO_2_ homeostasis and is not correlated with changes in ventilation. Instead, the reduction of V_A_/Q matching was proposed as the cause, due to the release of hypoxic pulmonary arterial vasoconstriction, with a consequent increase in functional dead space ventilation [24,25,26,27,28]. However, in another study in COPD patients, oxygen-induced hypercapnia was characterized by reduction in ventilation; the increased dead-space ventilation in the group of patients where PCO_2_ increased more than 3 mmHg (retainers) was attributed to bronchodilation due to the higher CO_2_ tension [29]. Another mechanism implicates the Haldane effect, in which oxygen displaces the CO_2_ dissociation curve to the right, increasing PaCO_2_, which cannot be normalized as patients with severe COPD are unable to increase ventilation [25,30].

## 4. Metabolic acidosis

Non-anion gap (non-AG) metabolic acidosis as well as increased AG acidosis may occur in asthma (Table 3).

### 4.1. Non-Anion Gap Metabolic Acidosis

Although metabolic acidosis in asthma is often considered to be secondary to hyperlactatemia, non-anion gap (non-AG) metabolic acidosis has also been reported. The presence (and absence thereof) of non-AG in patients with acute asthma has been a subject of debate in medical literature, with many studies [3,4] reporting no such findings, and others [35,36] documenting simple or mixed metabolic acidosis in as many as 38% of the study population. Mountain et al. noted that metabolic acidosis in acute asthma was more likely to occur in male patients and in patients with greater airflow obstruction and lower FEV_1_ [31]. Rashid et al. studied the clinical outcome in 109 adult patients hospitalized for asthma exacerbations. These patients were divided in three groups: group I included those patients who did not present with metabolic acidosis, group II those with AG metabolic acidosis, and group III those with non-AG metabolic acidosis. Out of these, 32 (29.4%) developed non-AG metabolic acidosis, while metabolic acidosis with elevated anion gap occurred in 11 patients (10.1%) [32]. The group of patients with non-AG metabolic acidosis had significantly higher chloride anion (Cl^−^) concentrations, with a tendency to hyperchloremia, while sodium (Na^+^) and potassium (K^+^) concentrations were not different compared to patients without acidosis or with elevated AG acidosis. The patients with non-AG acidosis were also at increased risk of respiratory failure and need for invasive, mechanical ventilation. Another study evaluated the acid-base status in 22 patients with acute severe asthma [36]. Ten patients had metabolic acidosis, defined by a base deficit > 2 mEq/L. None of these patients had an elevated AG. The most likely explanation for the observed metabolic acidosis in these patients, as in the previous study, was the renal loss of bicarbonates (HCO_3_^−^) due to renal compensation for preexistent, sustained hypocapnia. The HCO_3_^−^ levels were low-normal or decreased in these patients, while the pH values ranged from alkaline to moderately acidotic. It appears that non-AG acidosis is a frequent acid-base disorder in asthmatic exacerbations, due to the excretion of HCO_3_^−^, in response to chronic, sustained hypocapnia. Hypocapnia immediately shifts the dissociation reaction of H_2_CO_3_ to the left

CO_2_ + H_2_O ↔ H_2_CO_3_ ↔ H^+^ + HCO_3_^−^ (1)

reducing H^+^ and HCO_3_^−^ concentrations and increasing pH. In the short term, the decrease in PaCO_2_ alkalinizes the extracellular fluid, to the extent predicted by the Henderson–Hasselbalch equation. Persistent hypocapnia causes a further decrease in HCO_3_^−^ concentration, due to suppression of renal acid secretion/increase of HCO_3_^−^ excretion [37,38]. By definition, the primary metabolic acidosis of this type, i.e., with non-elevated AG, is accompanied by an increase in the concentration of Cl^−^, i.e., hyperchloremic acidosis [39]. Hyperchloremic, non-AG acidosis, secondary to chronic hypocapnia, was also noted in the study of Rashid et al. [32], in asthmatic patients. However, in an earlier animal study it was found that, during compensation for hypocapnia, the reduction in renal proton excretion was associated with increased Na^+^ excretion and not with Cl^−^ retention and hyperchloremia [40]. Furthermore, in another study, chronic hypocapnia was found to suppress renal acid secretion while inducing renal K^+^ wasting [41]. Interestingly, in a recent study [42], ascent to high altitude elicited a hypoxic ventilatory response in normal adults, resulting in increased ventilation and decreased PaCO_2_; hypocapnia was accompanied by strong ion difference (SID) reduction, signaling metabolic acidosis according to Stewart’s view [43]. Concerning the non-AG metabolic acidosis in patients with asthma, as a compensatory response to chronic hypocapnia, we should comment on the following issues:

1. Insofar as chronic hypocapnia in these patients is accompanied by hyperchloremia, the role of Cl^−^ channels in vascular and non-vascular smooth muscle contraction, as in the human airways, must be stressed, e.g., an alteration of Cl^−^ concentration changes the myogenic tone in the blood vessels [44,45]. Additionally, Cl^−^ channels in epithelial cells may affect mucus hydration on the airway surfaces [46]. Overall, Cl^−^ may have a critical role in asthma pathophysiology.
2. In conditions like asthma exacerbations, where an acute acid-base disorder complicates a chronic respiratory disorder, such as chronic hypocapnia in asthmatics, the use of base excess (or base deficit) method [47] to assess the severity of metabolic acidosis can lead to ‘erroneous assessment’ of the patient’s acid-base status, lacking any clinical relevance. Thus, in the study of Okrent et al. [36], in patients with acute severe asthma, metabolic acidosis was diagnosed by the increase of base deficit > 2 mEq/L. In this study, authors supported that this indicated a true loss of the body’s alkaline reserve. Nevertheless, one of the patients with the more severe metabolic acidosis, diagnosed with the base excess criterion (−4.9 mEq/L), had hypocapnia (PCO_2_ = 27 mmHg), pH higher than the mean physiological value (7.43), and HCO_3_^−^ concentration lower than the normal value (19 mEq/L), for which, however, no treatment is indicated, and which actually corresponds to the expected metabolic compensation for a chronic respiratory alkalosis (the expected HCO_3_^−^ concentration reduction (Δ(HCO_3_^−^)) equals 0.4 × ΔPCO_2_, i.e., (HCO_3_^−^) = 18.8 mEq/L) [37]. Thus, the physiologic compensation for an uncomplicated acid-base disturbance has been viewed as a serious metabolic acidosis superimposed on the chronic respiratory disorder. Overall, caution is needed in assessing the metabolic component of these acid-base disorders by utilizing the base excess values; diagnostic errors and therapeutic ill-practices may occur when they are not considered alongside the required clinical information. Criticism on the subject has long been made by Schwartz and Relman [48], which even took the form of a ‘transatlantic debate’ with arguments from both sides [49].
3. Finally, regarding the increased clinical risk demonstrated in asthmatics with non-AG acidosis (accompanied by hyperchloremia) [32], it should be noted that there are several studies suggesting that hyperchloremia per se is associated with poor outcome in hospitalized and critically ill patients [50,51,52]. Hyperchloremia induced by intravenous administration of crystalloid solutions with high Cl^−^ concentration is not to be overlooked [53,54], although there is no study investigating this issue exclusively in patients with acute severe asthma. In addition, hypocapnia, besides the acid-base balance, can have serious effects on the organs and systems in the body, and can adversely affect outcome in the critically ill [55].

### 4.2. Lactic Acidosis

Hyperlactatemia is a very common finding in patients with acute severe asthma. Lactic acidosis has two types: type A, which is associated with impaired oxygen delivery; and type B, where the oxygen delivery is normal but cellular function is impaired. The exact cause of lactic acidosis in these patients remains elusive, and several possible mechanisms have been proposed over the years. Acute severe asthma is characterized by hypoxemia [3,56] and may be accompanied by functional cardiac disorders (heart-lung interaction); increased right ventricular (RV) afterload during acute severe asthma, with increased impedance to RV ejection, and reduction of left ventricular (LV) preload and LV compliance due to leftward septal shift may reduce cardiac output [57,58]. Thus, lactate increase may result from severe hypoperfusion and decrease in oxygen supply to the tissues. In addition, respiratory muscle fatigue, due to increased respiratory muscle work load, can also increase lactate levels [59,60], especially under hypoxic conditions along with compromised tissue perfusion. In patients with acute severe asthma, a negative correlation between the peak lactate levels and the phosphate levels on admission was found [33]. In this study, hypophosphatemia preceded the increase in lactate levels in most patients. Hypophosphatemia may complicate treatment with bronchodilators in acute severe asthma patients [61]. Low phosphate levels in the blood may lead to reduced ATP synthesis in the muscles, accounting for muscle weakness and respiratory and heart failure [62]. Additionally, hypophosphatemia is known to impair the contractile properties of the diaphragm [63] and increase the hemoglobin affinity for oxygen due to a decrease in 2,3-diphosphoglycerate (2,3-DPG) [64], compromising tissue oxygenation. In a state of low cardiac output, hepatic perfusion decreases; also, the RV function disorder can increase the right heart filling pressures and lead to hepatic congestion [65,66,67]. Thus, hepatic dysfunction during acute severe asthma may result in impaired lactate clearance and hyperlactatemia [68,69]. An increase in lactate levels can also be observed during clinical improvement after bronchodilator treatment. It has been suggested that reperfusion of the previously ischemic organs, e.g., respiratory muscles, can lead to lactate release [70,71]. That is, the increase in lactate intracellular levels during the period of ischemia and anaerobic respiration may not be reflected in the lactate circulating levels in the serum. Serum lactate concentration can be increased after restoring tissue perfusion. Increased catecholamines in plasma may increase metabolic rate and lactate production without coexisting cell hypoxia. Increased levels of catecholamines in the blood have been found in patients with asthma, especially norepinephrine [72]. Additionally, catecholamines are used therapeutically during acute severe asthma, to promote bronchodilatation [73] and/or hemodynamic support. However, catecholamines have marked metabolic effects and may cause hyperlactatemia [74]. Treatment with bronchodilators has also been implicated in the lactate increase, i.e., the use of β_2_-adrenergic agonists, such as salbutamol [75]. In animals, β_2_-adrenoreceptor stimulation after salbutamol administration induced a significant increase in plasma lactate concentration; lactate increase was inhibited by clonidine (α_2_-adrenoreceptor agonist), a drug with opposite effects on the system of adenylate cyclase [76]. Stimulation of β_2_-adrenergic receptors increases glycogenolysis in the liver and muscle as well as lipolysis, through the increase of intracellular cAMP [77]. Free fatty acids liberated during lipolysis inhibit the oxidation of pyruvate by pyruvate dehydrogenase and may further increase lactate production [78]. Finally, theophylline and glucocorticoids may have a role in the increased lactate production during acute severe asthma. Theophylline is a non-selective 5’-phosphodiesterase inhibitor and potentiates the activity of β-adrenergic agents by increasing the intracellular concentration of cAMP [79,80,81]. Glucocorticoids are also known to increase the β-receptor’s sensitivity to β-adrenergic agonists [82]. Thus, when treating severe asthma attack, despite improvement in bronchospasm, a patient may hyperventilate and look more dyspneic; this may be a compensatory mechanism for lactic acidosis induced by therapy, to maintain pH within normal limits, and should not be seen as a worsening of airway obstruction [83]. In order to distinguish the type of lactic acidosis (A and B), the ratio of the concentration of lactate to the concentration of pyruvate in the blood can be used. Under aerobic conditions, this ratio is normally low, whereas under anaerobic conditions, due to the inability of cells to further metabolize pyruvate in mitochondria, this ratio increases to levels > 25:1 [84,85]. Thus, in a study concerning children with asthma, lactic acidosis was found to be predominantly of type B, with normal oxygen supply to the tissues, and was attributed to β-adrenergic stimulation [34].

## 5. Conclusions

Various acid-base disorders, of complex etiology, have been observed in asthma. Airway hyperresponsiveness leads to hyperventilation and chronic hypocapnia with a consequent increase in renal bicarbonate loss. This results in hyperchloremic acidosis, which becomes more clinically evident-with a clear effect on blood acidity-during severe asthma attacks, in case PCO_2_ normalizes or increases. Hypocapnia, and possibly hyperchloremia, may be related to the pathogenicity of the disease. Hypercapnia characterizes severe asthma attacks, with imminent risk for intubation and mechanical ventilation. Hypercapnia has been attributed to both the severity of the functional respiratory disorder and treatment with high oxygen mixtures (hyperoxia induced). Finally, increased AG metabolic acidosis has also been observed in asthma, i.e., lactic acidosis. Its etiology includes: **a.** disturbance of tissue oxygenation, e.g., by cardiac output reduction and hypoxemia or increased oxygen demands of respiratory muscles due to increased workload, **b.** reduced lactate clearance due to liver congestion and dysfunction, and **c.** treatment effect, e.g., β-agonists, on cellular metabolism, resulting in increased lactate production. The thorough and careful evaluation of acid-base disorders in asthma will serve the differential diagnostic approach concerning the underlying pathogenetic disorder and its treatment.

## Figures and Tables

**Table 1:** Respiratory alkalosis.

Study	Study Design	Study Population	Methods	Significant Findings
Osborne C.A. et al., 2000 [6]	Case-Control Study	23 asymptomatic asthmatics, 17 healthy subjects	Measured various stable state parameters	PaCO_2_ and P_ET_CO_2_ lower in asymptomatic asthmatics
Van den Elshout et al., 1991 [7]	Case- Control Study	30 asthmatics, 17 healthy subjects	Induction of hypercapnia and hypocapnia	Hypocapnia induced increases in airway resistance in asthmatic patients
Raimondi et al., 2013 [8]	Case series	314 patients admitted for ASA	ABGs, electrolytes and spirometry results documented	Hypocapnia was prominent in less severe asthma exacerbations

Abbreviations used: ASA = acute severe asthma, ABGs = arterial blood gases, PETCO_2_ = end-tidal carbon dioxide.

**Table 2:** Respiratory acidosis.

Study	Study Design	Study Population	Methods	Significant Findings
Mountain et al., 1988 [14]	Retrospective	61 patients with hypercapnic ASA, 168 with nonhypercapnic ASA	Various outcomes documented	Hypercapnic patients had more severe airway obstruction, symptoms
Lee K.H. et al., 1997 [15]	Retrospective	48 patients with 49 admissions to the ICU due to ASA	Various outcomes documented	Respiratory acidosis linked to higher mortality
Raimondi et al., 2013 [8]	Case series	314 patients admitted for ASA	ABGs, electrolytes and spirometry results documented	Inverse correlation between FEV_1_ and respiratory acidosis. Inability to perform spirometry linked to high pCO_2_
Cham et al., 2002 [16]	Prospective observational	127 patients with severe exacerbation of asthma and COPD in the ED	Acute respiratory acidosis documented and linked to clinical presentation	Drowsiness linked to sevenfold likelihood of respiratory acidosis. Flushing and intercostal retractions good predictors of respiratory acidosis

Abbreviations used: ICU = intensive care unit, COPD = chronic obstructive pulmonary disease, pCO_2_ = partial carbon dioxide pressure, ED = emergency department.

**Table 3:** Metabolic acidosis.

Study	Study Design	Study Population	Methods	Significant Findings
Mountain, R.D. et al., 1990 [31]	Retrospective	229 acute asthma episodes in 170 patients (Hospital Admissions)	Clinical features and arterial blood gases examined	Simple or mixed metabolic acidosis in 28% of the episodes.
Rashid, A.O. et al., 2008 [32]	Retrospective	109 patients hospitalized for asthma exacerbations	Acid-base, electrolyte status and outcomes	10.1% AG acidosis, 29.4% NAG acidosis. NAG acidosis patients had significantly higher intubation rates
Rabbat, A. et al., 1998 [33]	Prospective	29 non-intubated patients admitted to the ICU for ASA	Serial lactate measurements during treatment, correlation with outcomes	Hyperlactatemia a common finding on admission (59%) or during treatment (100%). No prognostic value, no correlation with PaCO_2_ or PEF
Meert, K.L. et al., 2012 [34]	Prospective observational	105 children with ASA admitted to a PICU	Blood lactate measurements followed by lactate/pyruvate ration measurements	Primarily type B lactic acidosis (associated with normal oxygen delivery). Presumed to be due to β-adrenergic stimulation
Raimondi et al., 2013 [8]	Case Series	314 patients admitted for ASA	ABGs, electrolytes and spirometry results documented	Most cases of metabolic acidosis attributed to chronic hypocapnia. Hyperlactatemia attributed mostly to adrenergic stimulation

Abbreviations used: AG = anion-gap, NAG = non-anion gap, PEF = peak expiratory flow, PICU: pediatric intensive care unit, PaCO_2_ = partial carbon dioxide tension.
